# Editorial: Advanced ophthalmic imaging in glaucoma and other optic neuropathies

**DOI:** 10.3389/fopht.2025.1629342

**Published:** 2025-06-11

**Authors:** Yukihiro Shiga, Takashi Nishida, Jin Wook Jeoung, Brad Fortune

**Affiliations:** ^1^ Neuroscience Division, Centre de Recherche du Centre Hospitalier de l’Université de Montréal, Montréal, QC, Canada; ^2^ Department of Neuroscience, Université de Montréal, Montréal, QC, Canada; ^3^ Hamilton Glaucoma Center, Shiley Eye Institute, Viterbi Family Department of Ophthalmology, University of California, San Diego, San Diego, CA, United States; ^4^ Department of Ophthalmology, Seoul National University Hospital, Seoul National University College of Medicine, Seoul, Republic of Korea; ^5^ Discoveries in Sight Research Laboratories, Devers Eye Institute and Legacy Research Institute, Legacy Health, Portland, OR, United States

**Keywords:** glaucoma, optic neuropathies, optical coherence tomography (OCT), optical coherence tomography angiography (OCTA), magnetic resonance imaging (MRI), ophthalmic imaging techniques, artificial intelligence (AI), multiple imaging modalities

Glaucoma is a major cause of irreversible blindness, necessitating continuous improvements in imaging techniques for early detection and effective management. Recent advancements in optical coherence tomography (OCT), OCT angiography (OCTA), magnetic resonance imaging (MRI), and artificial intelligence (AI)-assisted analysis have enhanced diagnostic precision and monitoring of disease progression. This Research Topic highlights the latest developments in ophthalmic imaging and AI-based algorithms for glaucoma and other optic neuropathies, emphasizing key techniques that improve our understanding of structural and vascular changes in the eye.

Keller et al. present a comprehensive summary of advancements in high-resolution imaging techniques for the iridocorneal angle, including gonio-photography, OCT-based and non-OCT-based imaging, as well as AI-driven approaches. The iridocorneal angle, containing Schlemm’s canal and trabecular meshwork, plays a pivotal role in aqueous humour outflow and is fundamental to glaucoma classification and treatment strategy. This review synthesizes current evidence, identifies limitations, and discusses future directions for high-resolution imaging of the iridocorneal angle in glaucoma management.

Shiga et al. emphasize the utility of OCT and OCTA in the posterior segment of the eye for diagnosing glaucoma and detecting its progression. OCT is essential for diagnosing and monitoring glaucoma, enabling non-invasive and objective assessment of structural changes from the anterior to the posterior segment. More recently, OCTA has emerged as a technique for non-invasive assessment of vascular perfusion, including patency of the retinal capillary plexuses and microvasculature of the optic nerve head. This systematic review provides an update on OCT and OCTA as essential tools in glaucoma care. In addition, this review focuses on the importance of detecting macular damage in glaucoma, as well as the latest findings from optic nerve head imaging and future directions of the OCT and OCTA applications.

Jiang et al. developed improved data acquisition and analysis methods for MRI of layer-specific retinal and choroidal blood flow. They applied this approach to detect reduced ocular blood flow in a well-established mouse model of glaucoma in both eyes. MRI-based imaging has emerged as a promising technique for studying ocular blood flow, addressing the depth resolution limitations of conventional vascular imaging methods. MRI facilitates a more comprehensive analysis of neurovascular dynamics in glaucoma by enabling precise quantification of retinal and choroidal blood flow. This study introduces an MRI-based simultaneous acquisition of bilateral data by dual eye coil setup, along with a novel retinal blood flow analysis approach. These advancements may provide deeper insight into ocular blood flow changes in glaucoma *in vivo*.

Glaucoma, optic neuritis, and non-arteritic anterior ischemic optic neuropathy produce distinct retinal ganglion cell (RGC) damage patterns. Wang et al. used a booster Variational Autoencoder (bVAE), a deep learning model, to capture spatial variations in RGC loss in glaucoma and other optic neuropathies. The authors confirmed that the developed bVAE model could track spatial patterns of RGC thinning over time and classify underlying causes based on generated latent space montage maps, which visualize different degrees and spatial patterns of RGC axon bundle damage. The proposed approach using the bVAE model effectively captures key spatial features in the optic neuropathies and facilitates visualization and quantification of RGC thinning patterns, potentially aiding in predicting disease progression.

In summary, the articles collected in this Research Topic cover 1) technological innovations such as OCT-based imaging techniques in assessing the anterior and posterior structures of the eye, 2) advances in techniques to visualize and quantify ocular blood flow in glaucomatous eyes, and 3) AI algorithms applications to extract disease-specific features. This Research Topic offers a broader perspective on imaging technologies and analytical approaches that contribute to the early detection and monitoring of glaucoma, essential for disease management. Future research should prioritize the integration of multiple imaging modalities to provide a more comprehensive view of glaucoma pathophysiology. Establishing standardized imaging biomarkers and increasing accessibility to advanced imaging tools will further enhance glaucoma management, ultimately leading to better patient outcomes.

